# The NMDA receptor complex: a multifunctional machine at the glutamatergic synapse

**DOI:** 10.3389/fncel.2014.00160

**Published:** 2014-06-10

**Authors:** Xuelai Fan, Wu Yang Jin, Yu Tian Wang

**Affiliations:** Brain Research Centre and Department of Medicine, Vancouver Coastal Health Research Institute, University of British ColumbiaVancouver, BC, Canada

**Keywords:** NMDA receptors, protein-protein interaction, synaptic plasticity, dopamine receptors, excitotoxicity, ion channel complex, signaling pathways

## Abstract

The N-methyl-D-aspartate receptors (NMDARs) are part of a large multiprotein complex at the glutamatergic synapse. The assembly of NMDARs with synaptic proteins offers a means to regulate NMDAR channel properties and receptor trafficking, and couples NMDAR activation to distinct intracellular signaling pathways, thus contributing to the versatility of NMDAR functions. Receptor-protein interactions at the synapse provide a dynamic and powerful mechanism for regulating synaptic efficacy, but can also contribute to NMDAR overactivation-induced excitotoxicity and cellular damage under pathological conditions. An emerging concept is that by understanding the mechanisms and functions of disease-specific protein-protein interactions in the NMDAR complex, we may be able to develop novel therapies based on protein-NMDAR interactions for the treatment of brain diseases in which NMDAR dysfunction is at the root of their pathogenesis.

N-methyl-D-aspartate receptors (NMDARs) are a major subtype of glutamate-gated ion channels at the excitatory synapses in the central nervous system (CNS), which mediate the flow of sodium (Na^+^) and calcium (Ca^2+^) ions into the cell and potassium ions (K^+^) out of the cell. NMDARs are heterotetrameric plasma membrane channels composed of two obligatory GluN1 and two modulatory GluN2 (A-D) subunits (Cull-Candy et al., 2001; Collingridge et al., 2009), although sometimes the GluN2 subunits are replaced by GluN3(A-B) subunits (Ulbrich and Isacoff, 2008). NMDARs form a diheteromer when the two GluN2 subunits are identical, or a triheteromer when two different GluN2 subunits co-assemble with two identical GluN1 subunits (Ulbrich and Isacoff, 2008; Collingridge et al., 2010). At resting state, NMDARs are blocked by the presence of extracellular magnesium ions (Mg^2+^) in the channel pore (Vargas-Caballero and Robinson, 2004). As such, the activation and opening of NMDARs is both voltage-dependent and ligand-gated, and requires the binding of two ligands, glutamate and either D-serine or glycine (Nong et al., 2003; Papouin et al., 2012), at a depolarized membrane potential to relieve Mg^2+^ block. The function of NMDARs in the CNS has been extensively studied in both genetic and pharmacological manipulations. NMDARs play a critical role in a wide range of cellular processes and brain functions, including synaptic plasticity, addiction and stroke (Schilström et al., 2006; Collingridge et al., 2010; Lai et al., 2011). The versatility of NMDAR functions may in part be attributed to its organization at the synapse. NMDARs are anchored to the plasma membrane as a multiprotein complex by binding to more than 70 adhesion proteins (Naisbitt et al., 1999; Husi et al., 2000; Grant and O’Dell, 2001). Upon activation, Ca^2+^ influxes through the opened channel pore and triggers various intracellular signaling cascades by activating calcium-sensitive NMDAR-interacting proteins in the multiprotein complex (Lai et al., 2011; Martin and Wellman, 2011; Lisman et al., 2012). Increasing evidence suggest that it is these interacting proteins that confer the versatile functions of NMDARs. In this review, we will introduce several key NMDAR-interacting proteins in the NMDAR multiprotein complex and discuss their critical roles in mediating physiological and pathological functions in the brain.

## Interactions between NMDARs and calcium sensing proteins in synaptic plasticity

**Figure 1:**
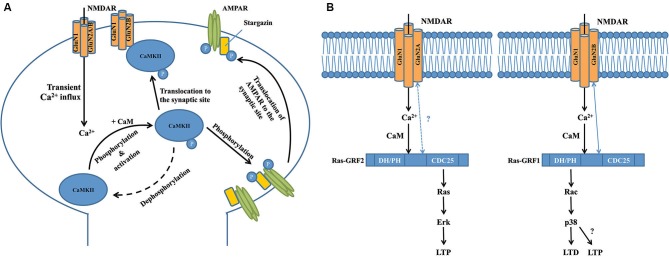
**Interactions between calcium-sensing proteins and NMDARs in the NMDAR complex and their critical roles in NMDAR-dependent synaptic plasticity. (A)** GluN2B-CAMKII interaction is required for the induction of long-term potentiation at the excitatory glutermaterigic synapse. At the postsynaptic domain, transient Ca^2+^ influx through the NMDAR induces autophosphorylation (at T286 on CAMKIIα subunits or T287 on CAMKIIβ subunits) of CaMKII, resulting in its persistent activation and subsequent translocation to the synaptic site where it binds to the GluN2B subunit of NMDAR. Activated CaMKII can phosphorylate the S831 residue of GluA1 subunit of AMPAR that significantly increases the single-channel conductance of the receptor. Meanwhile, activated CaMKII can also phosphorylate the postsynaptic scaffolding protein stargazin to facilitate the trafficking of AMPAR from the extrasynaptic space to the synaptic region so as to enhance synaptic transmission. **(B)** Transient calcium influx through GluN2B-containing NMDAR selectively activates Ras-GRF1 that contributes to LTD by activating the downstream Rac/p38 pathway, while calcium influx through GluN2A-containing NMDAR selectively activates Ras-GRF2 that contributes to LTP by activating the downstream Ras/ERK pathway. There is a selective physical interaction between Ras-GRF1 and the GluN2B subunit of NMDAR, but there is no evidence supporting the interaction between Ras-GRF2 and GluN2A subunit of NMDAR. Interestingly, a recent study showed that starting at 2 months of age in mice, Ras-GRF1 starts to contribute to the induction of LTP in the CA1 of hippocampus via the Rac/p38 pathway, however the exact mechanism is still unknown.

Two major forms of synaptic plasticity are long-term potentiation (LTP) and long-term depression (LTD), which are respectively characterized by long-lasting enhancement and reduction of synaptic transmission between two adjacent neurons after repetitive stimulation (Collingridge et al., 2010, 2004). Many proteins in the NMDAR complex contribute to these processes, with one of the most well-characterized proteins being Ca^2+^/calmodulin-dependent protein kinase II (CaMKII; Hayashi et al., 2000; Pi et al., 2010; Lisman et al., 2012), a serine/threonine protein kinase that is highly enriched in the post-synaptic density region. CaMKII is a large holoenzyme consisting of 12 identical subunits (Lisman et al., 2012). Transient Ca^2+^ influx through the NMDAR channel pore induces autophosphorylation of CaMKII (at T286 on CaMKIIα subunits or T287 on CaMKIIβ subunits), resulting in its persistent activation even after intracellular Ca^2+^ levels return to baseline (Rellos et al., 2010). As shown by a variety of real-time imaging studies and binding assays, activated CaMKII then rapidly and reversibly translocates to the spine and physically interacts with the 1260-1309aa domain in the carboxyl-terminal of the GluN2B NMDAR subunit (Figure 1A; Strack and Colbran, 1998; Bayer et al., 2001, 2006; Otmakhov et al., 2004; Zhang et al., 2008; Lisman et al., 2012). Although there is a basal level of association between CaMKII and NMDARs (Leonard et al., 1999), the translocation of these activated, phosphorylated CaMKII proteins greatly increases the total number of the kinase at the postsynaptic site. Introduction of a T286A mutation that prevents the autophosphorylation and activation of CaMKIIα in mice significantly impairs NMDAR-dependent LTP in the hippocampal CA1 area and memory performance in a Morris water maze task (Giese et al., 1998), suggesting that CaMKII plays a vital role in NMDAR-dependent synaptic plasticity. This is further supported by several other lines of evidence. For example, overexpressing a GluN2B carboxyl-terminal fragment (839-1482aa) that disrupts the physiological interaction between NMDAR/CaMKII leads to severe deficits in hippocampal LTP and spatial learning in transgenic mice (Zhou et al., 2007). LTP in organotypic hippocampal slices is impaired either by acute replacement of the synaptic GluN2B with GluN2A subunit that shows less binding affinity with CaMKII, or by expression of a mutant synaptic GluN2B subunit that markedly reduces the binding affinity with CaMKII (Barria and Malinow, 2005). Taken together, these results suggest that association of activated CaMKII and NMDARs may be a necessary step for NMDAR-dependent synaptic plasticity.

How the NMDAR-CaMKII interaction contributes to the production of LTP is still not fully understood. Several recent studies report that normal levels of LTP in the CA1 neurons in adult hippocampal slices can be induced after GluN2B-containing NMDARs are fully blocked, suggesting that functional activation of GluN2B-containing NMDARs may not be essential (Köhr et al., 2003; Liu et al., 2004; Woo et al., 2005; Foster et al., 2010). In this regard, it is interesting to note that a recent study has found that GluN2B in the NMDAR complex may function as a key scaffolding protein at excitatory synapses, and thus plays a critical role in LTP by recruiting molecules important for LTP through interacting with them via its cytoplasmic tail (Foster et al., 2010). Thus, it is likely that GluN2B may have a structural, rather than a functional, role for LTP production, presumably through the GluN2B-CaMKII interaction to recruit CaMKII to the activated synapses, and CaMKII (as a calcium-dependent kinase) in turn playing an essential role in LTP induction; however, the mechanistic details have not yet been elucidated. Bath application of AC3-1, a selective peptide inhibitor for CaMKII, to acute hippocampal slices prevents LTP induction, but has little effect on NMDAR channel function (Chen et al., 2001). This suggests that although activated CaMKII is recruited to the synaptic site by interacting with the GluN2B carboxyl-terminal, it does not regulate NMDAR channel function during synaptic plasticity. Indeed, several other studies have reported that activated CaMKII phosphorylates the S831 residue of the GluA1 subunit of the alpha-amino-3-hydroxy-5-methyl-4-isoxazolepropionate glutamate receptor (AMPAR), a major glutamate receptor that mediates fast synaptic transmission, and increases the single-channel conductance of GluA1-containing AMPARs (Figure 1A; Barria et al., 1997; Derkach et al., 1999; Kristensen et al., 2011). This effect can be mimicked by mutating the S831 residue of GluA1 to the phosphomimic glutamate residue (Kristensen et al., 2011). Meanwhile, activated CaMKII can also phosphorylate stargazin (Tomita et al., 2005), an important postsynaptic scaffolding protein that facilitates the trafficking of AMPARs from the extrasynaptic space to the synaptic region (Figure 1A; Schnell et al., 2002; Tsui and Malenka, 2006; Opazo et al., 2010; but see Kessels et al., 2009). Taken together, these processes markedly enhance synaptic transmission so as to promote the expression of LTP, in particular the initial phase of LTP. Given that LTP can exist for hours or even weeks, yet CaMKII is generally inactivated in a relatively short timeframe (∼1 min) after transient synaptic stimulation (Lee et al., 2009), it is still unclear whether CaMKII plays any important roles in the maintenance of late-phase LTP. Furthermore, little is known about how activated CaMKII participates in LTD, the opposing form of synaptic plasticity to LTP, although several recent studies have suggested that autonomous CaMKII can lead to either LTP or LTD, depending on the phosphorylation state of the control point, T305/T306 (Pi et al., 2010; Coultrap et al., 2014). Answering these questions will further uncover details about the physiological functions of GluN2B-CaMKII interaction in the NMDAR multiprotein complex, and enhance our understanding of NMDAR-dependent synaptic plasticity.

Two other important calcium sensors in the NMDAR multiprotein complex are Ras-Guanine Nucleotide-Releasing Factor 1 (Ras-GRF1) and Ras-GRF2, a family of calcium-dependent guanine nucleotide exchange factors (GEF) that are predominantly expressed in adult CNS neurons (Feig, 2011). Structurally, Ras-GRF1 and Ras-GRF2 share many similarities, and contain several common functional domains, including the calmodulin-binding IQ domain, Ras GTPase–activating CDC25 domain and Rac GTPase-activating DH/PH domain (Feig, 2011). However, recent studies have shown that Ras-GRF1 and Ras-GRF2 interact with different NMDAR subunits and play strikingly different roles in NMDAR-dependent synaptic plasticity (Figure 1B; Feig, 2011). Knocking out Ras-GRF1 in mice shows minimal effects on both high frequency stimulation (HFS) and theta burst stimulation (TBS)-induced LTP in hippocampal slices, but leads to severe impairments in low frequency stimulation (LFS)-induced LTD (Li et al., 2006). In contrast, knocking out Ras-GRF2 significantly impairs both HFS and TBS-induced LTP in hippocampal slices, whereas LFS-induced LTD is not affected. More strikingly, as shown by immunoblotting studies in hippocampal brain slices, Ras-GRF2 mediates signaling from GluN2A-containing NMDARs to the Ras effector extracellular signal-related protein kinase 1/2 (Erk1/2) mitogen-activated protein (MAP) kinase, a promoter of LTP, whereas Ras-GRF1 mediates signaling from GluN2B-containing NMDARs to the Rac effector p38 MAP kinase, a promoter of LTD (Figure 1B; Li et al., 2006). Given that some evidence suggests that GluN2A-containing NMDARs promote LTP whereas GluN2B-containing NMDARs promote LTD (Liu et al., 2004; Massey et al., 2004), these distinct differences between Ras-GRF1 and Ras-GRF2 in synaptic plasticity may be partially explained by the selective interaction between Ras-GRF1 and the 886-1310aa domain in the carboxyl-terminal of GluN2B subunit (Krapivinsky et al., 2003). However, to date there is no clear evidence that supports the presence of a direct interaction between Ras-GRF2 and the GluN2A subunit, although it is suggested that Ras-GRF2 may localize in the vicinity of GluN2A-containing NMDARs at the postsynaptic site (Jin and Feig, 2010). Furthermore, given that both Ras-GRF1 and Ras-GRF2 contain the activation domains for both Ras and Rac GTPases, it is also important to determine how and why these two proteins are coupled with different downstream signaling cascades during LTP and LTD. One explanation is that the selective association between the synaptic Ras GTPase-activating protein SynGAP and GluN2B-containing NMDARs in the synapse may help inhibit Ras-GRF1 from activating the Ras/Erk signaling cascade (Kim et al., 2005). Alternatively, scaffolding proteins that selectively associate with either GluN2A or GluN2B may specifically target Ras-GRF2 and Ras-GRF1 to the Ras and Rac signaling cascades, respectively (Buchsbaum et al., 2002; Feig, 2011). However, the detailed mechanism has yet to be determined.

It is noteworthy that the contribution of Ras-GRFs to NMDAR-dependent synaptic plasticity is highly regulated by development in mice. Ras-GRF1 and Ras-GRF2 are only coupled to NMDARs in adult neurons beginning at 20 days of age in mice (Li et al., 2006), while the guanine nucleotide exchange factor Sos mediates NMDAR signaling in neurons derived from neonatal mice (Tian et al., 2004). As mentioned above (Figure 1B), previous studies report that beginning at 1 month of age, Ras-GRF1 preferentially mediates GluN2B-containing NMDAR-dependent LTD in the CA1 region of the hippocampus of mice via the Rac/p38 pathway (Li et al., 2006). Interestingly, beginning at 2 months of age in mice, the role of Ras-GRF1 dramatically shifts to support the induction of LTP, rather than LTD, in the CA1 region as a downstream effector of calcium-permeable, AMPA-type glutamate receptors (Jin et al., 2013). Surprisingly, this induction of LTP is also mediated by the Rac/p38 pathway, which was previously thought to be mainly associated with LTD (Figure 1B; Jin et al., 2013). It is still unclear how these signaling pathways switch during development, but further investigation is likely to reveal additional details on the regulatory mechanisms of Ras-GRF in synaptic plasticity at the NMDAR complex.

## Interactions between NMDARs and G-protein coupled receptors (GPCRs) in psychiatric disorders

In addition to coupling to calcium sensing proteins, the NMDAR complex has extensive functional interactions with G-protein coupled receptors (GPCRs) through direct physical interaction. An example of this is the reciprocal modulation between NMDARs and dopamine receptors, a family of GPCRs that has been implicated in many psychiatric disorders, including schizophrenia (Seeman, 1987; Dolan et al., 1995).

**Figure 2:**
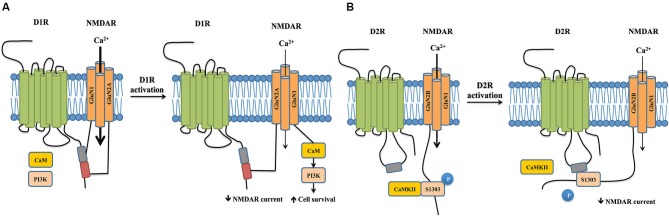
**Crosstalks between dopamine and NMDA receptors mediated by physical interactions between the two receptors**. **(A)** Physical interaction between D1R and NMDAR reciprocally regulate receptor properties and trafficking. The carboxyl tails of NMDAR subunits GluN1 and GluN2A individually binds to separate sites on the D1R carboxyl tail. D1R activation inhibits NMDAR currents through the physical interaction between GluN2A and D1R by reducing the surface number of NMDARs, which appears to impact working memory. In contrast, activation of D1R promotes the dissociation of GluN1 and D1R. This allows the recruitment of CaM and PI3K to GluN1, which activate PI3K-dependent cell survival signals and promote cell survival. **(B)** The carboxyl tail of GluN2B binds to the third intracellular loop of the D2R. Activation of D2R promotes its association with GluN2B, which in turn disrupts the binding between GluN2B and CaMKII. This leads to a decrease in CaMKII activity, resulting in reduced phosphorylation of Serine1303 of GluN2B and hence reduced NMDAR currents.

Dopamine receptors comprise of five subtypes, D1R to D5R, which can be further classified pharmacologically into D1-like receptors consisting of D1R and D5R, and D2-like receptors consisting of D2R, D3R and D4R (Seeman, 1987; Tiberi et al., 1991; Dolan et al., 1995). Although earlier studies have shown presynaptic localization (Levey et al., 1993), it is now generally believed that D1-like receptors are strictly localized on cells that are postsynaptic to dopaminergic neurons (Hersch et al., 1995; Yung et al., 1995). In contrast, D2Rs and D3Rs are present both presynaptically and postsynaptically (Sokoloff et al., 2006). D1-like receptors increase intracellular cAMP concentrations through activation of the G_s/olf_ class of G proteins and subsequent activation of adenylyl cyclase (AC), whereas D2-like receptors couple to the G_i/o_ class of G proteins to inhibit AC (Kebabian et al., 1972; Monsma et al., 1990). Numerous studies have demonstrated extensive crosstalk between DRs and NMDARs via direct physical association between the receptors (Figure 2, for a review, see Beaulieu and Gainetdinov, 2011).

D1Rs co-immunoprecipitates with both GluN1 and GluN2A subunits of the NMDAR in rat hippocampal tissue, suggesting a physical interaction between the two receptors (Lee et al., 2002). Further *in vitro* experiments confirm that the carboxyl tails of both the GluN1 subunit and GluN2A subunit (but not GluN2B) individually bind to the carboxyl tails of D1Rs (but not that of D5Rs). The 387-416aa domain of the D1R carboxyl-tail is sufficient for D1R-GluN1 binding, while the 417-446aa domain is required for the D1R-GluN2A interaction, suggesting that two distinct protein-protein interactions exist between the receptors. The binding between NMDARs and D1Rs occurs in the absence of D1R agonists, and D1R activation reduces the interaction between D1R-GluN1 but not that between D1R-GluN2A (Lee et al., 2002).

The functional consequences of these two interactions significantly differ. Following D1R stimulation with its agonist SKF81297, the D1R-GluN2A interaction functions to reduce the surface expression of NMDARs and hence the receptor-gated currents in both transfected cells and hippocampal neurons through a PKA/PKC independent pathway (Lee et al., 2002). It should be noted that SKF81297, in addition to activating D1Rs, has previously been shown to reduce NMDAR currents via a direct blockade of the NMDAR channel pore (Figure 2A, Cui et al., 2006). However, dissociation of D1R-GluN2A interaction using an interference peptide significantly reverses the attenuation of NMDAR currents, strongly arguing for a crucial role of the physical coupling between the two receptors, rather than a direct channel blockade, in mediating D1R activation-induced NMDAR inhibition (Lee et al., 2002). As overactivation of NMDAR is crucial for excitotoxic neuronal death (Lai et al., 2011), this reduction of surface expression of NMDARs would be expected to decrease Ca^2+^ influx and hence be neuroprotective. However, peptide-mediated dissociation of D1R-GluN2A does not affect neuronal survival following a NMDA insult. Instead, the uncoupling of D1R-GluN1 following D1R activation confers neuroprotection through the recruitment of calmodulin and phosphatidylinositol 3-kinase to GluN1, which activates cell-survival signals (Figure 2A, Lee et al., 2002). These data suggest that D1R activation-induced inhibition of NMDAR-mediated currents and excitotoxicity are differentially mediated by distinct subunit-specific interactions in the NMDAR complex, further highlighting that specific protein-protein interactions dictate the functional outcomes of the NMDAR complex.

Further studies have demonstrated that D1R and NMDAR interactions may also reciprocally regulate receptor trafficking and surface expression in a NMDAR subunit-specific manner. D1Rs directly interact with the GluN1 subunit in cytoplasmic compartments, where they are retained, and the presence of the GluN2B subunit drives the translocation and insertion of the D1R-GluN1 receptor complex into the plasma membrane in medium spiny neurons and cotransfected cells (Fiorentini et al., 2003). This suggests that D1Rs and NMDARs are assembled as constitutive heteromeric complexes in cytoplasmic compartments prior delivery to functional sites, a process that does not depend on receptor activation (Fiorentini et al., 2003). Furthermore, constitutive association with GluN2B-containing NMDARs abolishes agonist-induced D1R internalization and stabilizes D1Rs at the post-synaptic density (Fiorentini et al., 2003). In contrast, although GluN2A-containing NMDARs can also recruit D1Rs to the cell surface, this effect depends on NMDAR stimulation. In cotransfected cells, activation of GluN2A-containing NMDARs increases their physical association with D1Rs, which drives the insertion of D1Rs into the plasma membrane (Pei et al., 2004). These differences in agonist dependency may perhaps be attributed to functional differences between GluN2A or GluN2B-containing NMDARs (for a review, see Traynelis et al., 2010). In addition to regulating D1R surface expression, NMDAR activation can also reduce D1R lateral diffusion in medium spiny neurons via increased GluN1-D1R interaction, which stabilizes D1Rs at the synapse (Scott et al., 2006). It should be noted, however, that the observed increase in D1R surface levels may be transient or restricted to certain neuronal populations *in vivo*, as a 90% reduction in GluN1 expression in mice did not impair striatal D1R pharmacology and function (Ramsey et al., 2008).

Conversely, the binding between D1Rs and NMDARs also modulates NMDAR surface dynamics at glutamatergic synapses, which offers a more direct means to regulate synaptic plasticity. D1R activation, which reduces D1R-GluN1 interaction at the perisynapse, allows NMDARs to laterally diffuse into the post-synaptic density where they favor LTP, an effect that was recapitulated by dissociating D1R-GluN1 binding with an interference peptide (Ladepeche et al., 2013). Dissociation of D1R-GluN1 upon D1R activation promotes CaMKII-GluN1 interaction and increases CaMKII activity, which in turn upregulates NMDAR-mediated LTP in primary hippocampal neurons and promotes spatial working memory in the delayed match-to-place version of the water maze in intact animals (Nai et al., 2010). While this effect may be cautiously interpreted as a result of physical dissociation between D1R-GluN1, it should be noted that D1R activation may also affect synaptic plasticity through protein phosphatase-dependent pathways (Frey et al., 1993; Stramiello and Wagner, 2008) as well as network mechanisms (Xu and Yao, 2010). However, the relative contribution of each mechanism remains to be seen.

The long-form D2R, which is preferentially involved in postsynaptic signaling compared to short-form D2R (Lindgren et al., 2003) interacts with the carboxyl-tail of the GluN2B subunit of NMDARs in the post-synaptic density of striatal neurons. In contrast to D1Rs, this interaction is not mediated by the D2R carboxyl-tail, but by a TKRSSRAFRA motif situated in the N-terminal of the third intracellular loop of D2Rs (Liu et al., 2006). The interaction between D2Rs and NMDARs is receptor- and subunit- specific: D3Rs, which are similar to D2Rs and also belong to the D2-class of dopamine receptors, do not associate with the GluN2B subunit, and D2Rs do not interact with the GluN1 subunit (Liu et al., 2006).

Activation of D2Rs with its agonist quinpirole inhibits NMDAR currents in acutely dissociated medium-sized striatal neurons, which can be blocked by disrupting D2R-GluN2B physical interaction (Liu et al., 2006). Acute treatment with cocaine, a psychostimulant known to exert its effects through both dopaminergic and glutamatergic signaling, enhances D2R-GluN2B coupling in the striatum. In turn, this increased association disrupts GluN2B-CaMKII binding, resulting in decreased CaMKII activity, reducing phosphorylation of GluN2B at the Serine 1303 residue, and thereby resulting in reduced NMDAR currents (Figure 2B, Liu et al., 2006). Cocaine-enhanced D2R-GluN2B interaction seems to be specific to the striatum, as it was not observed in the hippocampus or frontal cortex. The D2R-GluN2B interaction may play a role in eliciting the locomotor effects of cocaine. In rats, systemic disruption of D2R-GluN2B interaction prior cocaine treatment markedly reverses the uncoupling between GluN2B-CaMKII induced by cocaine and rescues GluN2B S1303 phosphorylation in the striatum without affecting basal GluN2B-CaMKII interactions. Furthermore, blocking D2R-GluN2B interactions significantly, though not completely, reduces cocaine-stimulated locomotion in rats (Liu et al., 2006). This suggests the existence of other factors that contribute to the full-scale motor response to cocaine.

Going forward, it would be particularly interesting to see whether these findings extend to prefrontal dopaminergic neurotransmission. The prefrontal cortex, together with other corticolimbic areas including the cingulate gyrus and hippocampus, are implicated as part of the dysfunctional network that underlies schizophrenia (Fletcher, 1998). Furthermore, NMDAR hypofunction during development is gradually emerging as a convergence point for disease progression in schizophrenia (Snyder and Gao, 2013). Together, these findings, with the observation that the D1R-GluN1 physical interaction reciprocally regulates D1R and NMDAR surface expression and function, prompts the question of whether it is the initial failure in glutamatergic signaling that leads to reduced dopaminergic neurotransmission in schizophrenia through perturbed protein (receptor)-NMDAR interactions in the NMDAR complex.

In summary, NDMARs and dopamine receptors directly interact to reciprocally regulate receptor surface expression, channel properties and downstream intracellular signaling cascades. Together with functional interactions through shared downstream signaling molecules, the physical coupling between NMDARs and dopamine receptors adds another layer of regulation to both neurotransmitter systems to fine-tune neuronal function and behavior.

## NMDAR complex as a crux in ischemic neuronal damages

Intensive investigations into the mechanisms and functions of protein-protein interactions in the NMDAR complex have not only further advanced our understanding of the roles of NMDARs in brain function and dysfunction, but also led to the development of novel protein-NMDAR interaction-based therapeutics for treating brain disorders in which NMDAR dysfunction is at the root of their pathogenesis. Well-characterized examples of such therapeutics are the newly developed and promising interventions that protect neurons against excitotoxic/ischemic damages following stroke by disrupting direct or indirect interactions between NMDARs and neuronal death signaling molecules, such as neuronal Nitric Oxide Synthase (nNOS; Aarts et al., 2002; Zhou et al., 2010), death associated protein kinase 1 (DAPK1; Tu et al., 2010; Fan et al., 2014) and PTEN (Zhang et al., 2013).

Overactivation of the NMDAR triggers rapid Ca^2+^ influx that could lead to excitotoxic neuronal death. This excitotoxicity is considered a common pathological step leading to neuronal loss in many brain disorders, from acute brain injuries such as stroke to chronic neurodegenerative diseases such as Huntington’s disease (Lai et al., 2014). Yet direct blockade of NMDARs has failed as a neuroprotective strategy in stroke, at least in part due to intolerable psychosomatic side effects as a result of blocking normal NMDAR function and/or relatively narrow windows for effective intervention (Ikonomidou and Turski, 2002; Lai et al., 2014). In an effort to overcome the shortcomings of NMDAR antagonists, scientists have focused on druggable protein-protein interactions in the NMDAR complex that specifically lead to neuronal death. These have led to not only the identification of several well-characterized cell death-promoting molecules that can form neuronal death signaling complexes downstream of NMDARs via either direct or indirect interactions with NMDAR subunits, but also the development of numerous promising interventions that protect neurons from brain insults by specifically disrupting these interactions (Lai et al., 2014).

**Figure 3:**
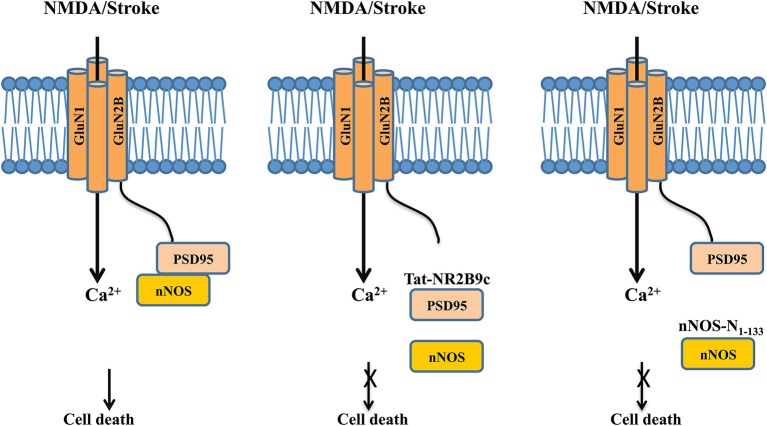
**Dissociating the NMDAR cell death signaling complex protects neurons against excitotoxic/ischemic damage following stroke. Left panel**: Following excitotoxic NMDA stimulation or ischemic insult, the post-synaptic scaffolding protein PSD-95 couples nNOS to the GluN2B subunit of the NMDAR, thus positioning nNOS for a more effective activation and production of NO by calcium influx through the NMDAR channel pore, leading to increased neuronal death. **Middle panel**: The interference peptide Tat-GluN2B9c competitively disrupts the interaction between PSD-95 and GluN2B, and hence dissociates PSD-95 and nNOS from the NMDAR complex. By doing so, the peptide reduces the NMDA-induced production of NO, and hence decreases NO-mediated excitotoxic/ischemic neuronal death. **Right panel**: Similarly, the interference peptide nNOS-N_1-133_ disrupts the binding between PSD-95 and nNOS, thereby dissociating nNOS from the NMDAR complex. By reducing the NMDAR-mediated activation of nNOS, the peptide reduces NO production and hence excitotoxic/ischemic neuronal death.

One of the most exciting bench-to-bedside examples is the recent development of effective neuroprotectants based on disrupting the GluN2B-Post synaptic density-95 (PSD-95)-nNOS cell death complex (Christopherson, 1999; Sattler et al., 1999), which was first characterized over a decade ago. PSD-95 is a membrane-associated guanylate kinase (MAGUK) concentrated at glutamatergic synapses and is involved in synapse stabilization and plasticity (El-Husseini et al., 2000). nNOS catalyzes the production of nitric oxide (NO), a diffusible signaling molecule implicated in synaptic plasticity (Bredt et al., 1990) and glutamate neurotoxicity (Dawson et al., 1991). By binding to both nNOS and GluN2B through its different PDZ domains, PSD-95 functions as a scaffolding protein to bring nNOS to the NMDAR complex (Kornau et al., 1995; Brenman et al., 1996). Following excitotoxic stimuli and/or ischemic insults, PSD-95 tethers nNOS to GluN2B, thus positioning nNOS to be more effectively activated by Ca^2+^ influx through the NMDAR channel pore and generation of the cytotoxic compound NO (Figure 3, left panel) (Dawson et al., 1991; Sattler et al., 1999).

In a proof-of-concept study, Aarts et al. (2002) disrupted this signaling complex with a 20-mer interference peptide Tat-NR2B9c, which comprises of the last nine amino acids of the carboxyl tail of GluN2B required for its interaction with PSD-95 and the 11-mer Tat protein transduction domain that renders the peptide plasma membrane permeable. The rationale behind the initial peptide design is to use it to disrupt GluN2B-PSD-95 interaction and hence to prevent PSD-95 from recruiting nNOS to the NMDAR complex, thereby reducing NO production and NO-mediated neuronal death (Figure 3, middle panel), although a later study suggests that the peptide may also somehow reduce the interaction between PSD-95 and nNOS (Cui et al., 2007). When bath applied to primary neuronal cultures and acute brain slices, Tat-NR2B9c does not affect NMDAR-mediated currents or Ca^2+^ fluxes, but can selectively disrupt the interaction between PSD-95 and NMDARs through competition with the binding of native GluN2B subunit (but not GluN2A) to PSD95, and thereby reduce the generation of NO and excitotoxicity (Aarts et al., 2002). When given systematically, a single dose of Tat-NR2B9c administered either before or after ischemic insults reduces ischemic brain damage with concurrent improvements in neurological scores in rats subjected to transient middle cerebral artery occlusion (MCAo), a well-characterized focal ischemia stroke model (Aarts et al., 2002). Similar neuroprotective effects are seen by disrupting the interaction between PSD-95 and nNOS with an interference peptide derived from the PSD-95 binding domain of nNOS (nNOS-N_1-133_; Figure 3, right panel; Zhou et al., 2010). Following viral infection of rats with a vector expressing this peptide, nNOS-N_1-133_ effectively blocks the interaction between the two proteins and significantly reduces stroke-induced ischemic damage (Zhou et al., 2010).

These initial attempts at targeting GluN2B-PSD-95-nNOS signaling pathway for the treatment of stroke was quickly followed by a well-designed study in a focal ischemia model in gyrencephalic nonhuman primates (Cook et al., 2012a,b) and a successful phase 2, double-blind, placebo-controlled multicenter clinical trial of neuroprotection in procedurally-induced stroke (Hill et al., 2012). Treatment with Tat-NR2B9c (also named as NA-1 in these studies) results in reduced ischemic brain damage compared to the placebo treatment, as evidenced by diffusion weighted magnetic resonance imaging. These exciting bench-to-bedside results provide great promises for further understanding disease-specific protein-protein interactions in the NMDAR complex and thereby developing novel and effective therapeutics for brain diseases involving NMDAR-mediated neuronal degeneration.

## Conclusions

Increasing evidence suggest that the NMDAR is not a solo player in the regulation of many brain functions and dysfunctions. By associating with various membrane receptors and extracellular or intracellular proteins in the complex, the NMDAR can contribute to various physiological processes such as learning and memory, and brain disorders such as stroke, schizophrenia and addiction. Many NMDAR-interacting proteins have recently been identified in the NMDAR complex; however, in the past only a few protein-protein interactions have been characterized in detail. Since NMDARs are widely expressed in all brain areas, it would be paramount to know whether and how the NMDAR plays vital roles in both brain function and dysfunction through these diverse protein-protein interactions in the receptor complex. Further investigation on this topic will not only deepen our understanding of the functions of the NMDAR multiprotein complex, but also greatly facilitate the development of innovative therapeutics in treating various NMDAR dysfunction-related brain disorders.

## Conflict of interest statement

The authors declare that the research was conducted in the absence of any commercial or financial relationships that could be construed as a potential conflict of interest.
